# Telemedicine for Elderly: A Pilot Study on Usability and Acceptability in Remote Cognitive Assessment

**DOI:** 10.1002/alz70857_103204

**Published:** 2025-12-25

**Authors:** Alexa Frederike Holfelder, Esther Brill, Michael Falkner, Christine R. Krebs, Rachid Guerchouche, Minh Tran‐Duc, Jacob Lahr, Stefan Klöppel

**Affiliations:** ^1^ University Hospital of Old Age Psychiatry and Psychotherapy, Bern, Bern, Switzerland; ^2^ ARTORG, University of Bern, Bern, Switzerland; ^3^ INRIA, Sophia Antipolis, France

## Abstract

**Background:**

With an aging population, cognitive impairments and dementia pose significant challenges. Accurate diagnosis and treatment are crucial, yet access to specialized dementia screening clinics remains limited, especially in rural and remote areas. Telemedicine presents a promising solution by offering accessible opportunities for cognitive testing. This pilot study examines the subjective experiences of elderly with cognitive decline using telemedicine for an initial dementia screening, emphasizing their perceptions and feelings toward digital technology, particularly in the context of remote cognitive assessments as a patient‐centered approach to dementia screening.

**Method:**

This ongoing pilot study utilizes telemedicine to conduct remote cognitive assessments via videoconferencing platform, which is specifically designed to support a full range of neuropsychological tests, facilitating the early detection of cognitive decline and monitoring cognitive performance. Data are collected to evaluate acceptability and user experience, focusing on the subjective perceptions of both participants and clinicians in remote memory clinic settings. Acceptability and user experience are measured using questionnaires scored on a Likert scale (1‐10) offering valuable insight into telemedicine experiences.

**Result:**

Participants (*n* = 10) reported high satisfaction scores for overall experience (8.33), video quality (8.39), and audio quality (8.19). In contrast, clinicians highlighted challenges, particularly with technical setup (4.13) and interaction quality (5.5). Data collection is expected to conclude by June 2025, providing a more comprehensive dataset for further analysis.

**Conclusion:**

Preliminary findings from this pilot study emphasize the potential of video‐supported remote diagnostics to expand access to dementia assessments in rural and underserved areas. High satisfaction ratings from participants demonstrate the feasibility of providing timely and comprehensive care while alleviating the need for long‐distance travel. However, challenges reported by clinicians, highlight the need for further refinements to improve usability and enhance clinician‐patient interactions. Future efforts should prioritize optimizing platform interactivity and streamlining the technical setup process to boost clinician satisfaction and minimize workflow disruptions. With these improvements, the system has the potential to address healthcare disparities in rural regions effectively.

